# Association between geriatric nutritional risk index and 28 days mortality in elderly patients with sepsis: a retrospective cohort study

**DOI:** 10.3389/fmed.2023.1258037

**Published:** 2023-09-26

**Authors:** Ling Li, Xiuhong Lu, Shuangwen Qin, Debin Huang

**Affiliations:** Department of Critical Care Medicine, The First Affiliated Hospital of Guangxi Medical University, Nanning, China

**Keywords:** geriatric nutritional risk index, mortality, sepsis, intensive care unit, MIMIC-IV

## Abstract

**Background:**

There is a significant controversy surrounding the impact of the geriatric nutritional risk index (GNRI) on mortality among elderly septic patients. This retrospective cohort study aimed to investigate the association between GNRI at admission and 28 days mortality in elderly septic patients.

**Methods:**

We retrospectively analyzed data collected from the MIMIC IV database between 2009 and 2019, which included 2,834 septic patients aged 65 years and above. The exposure variable was the GNRI, determined according to albumin levels, height, and weight. The primary outcome was 28 days mortality. We employed multivariable Cox regression analyses and Kaplan–Meier survival curves to examine the association between GNRI and 28 days mortality. We used restricted cubic splines to determine if there was a non-linear relationship between 28 days mortality and GNRI in elderly patients with sepsis and to examine the presence of a threshold saturation effect. In addition, interaction tests were conducted to identify subgroups that exhibited significant differences.

**Results:**

A total of 2,834 elderly patients with sepsis participated in the study. Following adjustment, multivariable Cox regression analyses demonstrated that the GNRI was related to 28 days mortality (HR = 0.97, *p* < 0.001, 95% CI: 0.97–0.98). An L-shaped connection between GNRI and 28 days mortality was discovered via restricted cubic spline analysis, with an inflection point of 98.1. On the left side of the inflection point, GNRI levels were significantly negatively linked with 28 days mortality (HR = 0.967, 95% CI: 0.959–0.974; *p* < 0.001), and on the right side, there was no significant correlation (HR = 1.043, 95% CI: 0.984–1.106; *p* = 0.1549).

**Conclusion:**

In this analysis of data from a large cohort of elderly septic patients, GNRI scores on admission were correlated with a 28 days risk of death from sepsis in the elderly suggesting that GNRI scores could serve as a valuable indicator for evaluating mortality rates among elderly septic patients in the intensive care unit (ICU).

## 1. Introduction

Sepsis is a severe, perhaps fatal condition caused by an aberrant host response to infection that leads to organ failure (1). Despite significant advancements in sepsis awareness and medical technology, sepsis remains a global concern, impacting millions of people annually, and resulting in mortality rates ranging from one-third to one-sixth of patients (2–4). In 2017, there were 489 million sepsis events globally, causing 110 million sepsis-related deaths, accounting for approximately 19.7% of global deaths. This highlights sepsis as a significant contributor to the worldwide disease burden (5). The incidence of sepsis among elderly patients has risen sharply due to the increasing aging population (6, 7), placing this demographic at a high risk of mortality. Importantly, sepsis is a leading cause of morbidity and death in older patients (8). Elderly patients, due to multiple factors such as advanced age, immune deficiency, and a prolonged host inflammatory response (9), are more prone to comorbidities and reduced functional reserve compared to younger adults. This complexity in the development of sepsis increases the risk of death (10, 11). Consequently, identifying high-risk patients in the ICU is crucial for reducing mortality rates among elderly septic patients.

The American Society for Parenteral and Enteral Nutrition (ASPEN) and the Society of Critical Care Medicine (SCCM) have recommended some nutritional screening tools, including the Critically Ill Patient Nutritional Risk (NUTRIC score) and the Nutritional Risk Screen-2002 (NRS 2002), to evaluate the nutritional status of critically sick patients because malnutrition is closely related to the prognosis of critically sick patients (12, 13). These evaluations, however, are carried out using subjective questionnaires that demand the patient’s involvement. They are not suitable for elderly critically sick patients and have limitations in their clinical application. Therefore, there is a need to identify a quick, simple, and objective tool that can assess the risk of malnutrition in critically sick elderly patients. The GNRI, created by Bouillanne et al. (14), is a straightforward tool for evaluating the nutritional condition of the elderly. It is computed using height, weight, and serum albumin. Numerous studies have shown that low GNRI scores are connected to poor prognosis in patients with heart failure (15), acute coronary syndromes (16), chronic hemodialyses (17), malignancies (18), and acute ischemic stroke (19). However, it is still unclear whether GNRI has clinical utility in predicting mortality in elderly septic patients.

Therefore, we retrospectively analyzed the association between GNRI and mortality in elderly septic patients using a large sample cohort from the MIMIC IV database.

## 2. Methods

### 2.1. Data sources

The MIT Computational Physiology Laboratory created and continues to maintain the MIMIC-IV (v2.2) database, which may be accessed by the general public at https://physionet.org/content/mimiciv/2.2/. From 2008 to 2019, the database covers data on critically ill patients admitted to Beth Israel Deaconess Medical Center (BIDMC). The dataset includes demographic details, vital signs, laboratory outcomes, medication treatments, and other clinical variables. To protect patient privacy, all personal data was de-identified and patient identifiers were removed, eliminating the need for patient consent or ethical approval. Fulfillment of the Collaborative Institutional Training Initiative (CITI) program and passing of the “Conflict of Interest” and “Data or Specimens Only Research” examines (ID: 12187573) were prerequisites for access to the database. LL, the primary author of this study, satisfactorily fulfilled these prerequisites and gained access to the database for extracting data. The STROBE (Strengthening the Reporting of Observational Studies in Epidemiology) statement is adhered to in the reporting of this study.

### 2.2. Study population

The MIMIC-IV database’s data for elderly sepsis patients who were admitted to the ICU were the focus of this investigation. Sepsis was defined as recommended by the Third International Consensus Definitions for Sepsis and Septic Shock (Sepsis-3), and had suspected or documented infection and a total Sequential Organ Failure Assessment (SOFA) score increase of at least 2. The criteria that followed were the inclusion requirements: (1) patients over the age of 65; (2) patients that made their first ICU admission (for patients who had numerous ICU admissions, only data from the first ICU admission for the initial hospitalization were included). The criteria that followed were the exclusion requirements: (1) patients having albumin, height, and weight lacking data; (2) patients with ICU stays of less than 24 h. In the end, 2,834 patients in total were involved in the study.

### 2.3. Data collection

The study extracted eligible patients from the MIMIC-IV database using Navicat Premium15 software and Structured Query Language (SQL). We retrieved or evaluated the variables, including (1) demographic information: age and gender; (2) comorbidities: myocardial infarction (MI), congestive heart failure (CHF), chronic obstructive pulmonary disease (COPD), acute kidney injury (AKI), diabetes, renal disease, malignant cancer, liver disease; (3) treatment: renal replacement therapy (RRT), mechanical ventilation (MV); (4) laboratory parameters: aniongap, blood urea nitrogen (BUN), red blood cell (RBC), white blood cell (WBC), platelets, hemoglobin, alanine transaminase (ALT), aspartate aminotransferase (AST), international normalized ratio (INR); (5) the scoring of organ dysfunction: sequential organ failure assessment (SOFA), simplified acute physiology score (SAPS II); (6) outcomes: 28 days mortality, hospital and ICU length of stay.

Based on the original description provided by Bouillanne et al. (14), the study population was classified into four risk groups based on the GNRI classification criteria: major risk group (GNRI <82; *n* = 728), moderate risk group (82 ≤ GNRI < 92, *n* = 1,034), low risk group (92 ≤ GNRI ≤ 98, *n* = 542), and no risk group (GNRI >98; *n* = 530). Use the following formula to calculate the GNRI:


$$\mathrm{GNRI}=1.489*\mathrm{albumin}\;\left(g/L\right)+\left[41.7*\left(\frac{\mathrm{weight}}{WLo}\right)\right]$$

The Lorentz formula, which was used to predict the ideal weight (WLo) based on height (*H*) and gender, is shown below:

For men:


$$WLo\;\left(\mathrm{kg}\right)=H\;\left(\mathrm{cm}\right)-100-\left[\frac{H-150}{4}\right]$$

For women:


$$WLo\;\left(\mathrm{kg}\right)=H\;\left(\mathrm{cm}\right)-100-\left[\frac{H-150}{2.5}\right]$$

When the actual weight exceeded WLo, weight/WLo was represented as 1.

### 2.4. Outcome variables

The primary outcome was 28 days mortality. Secondary outcomes included length of ICU stay and length of hospital stay. The 28 days mortality data were verified by looking through the database’s death records.

### 2.5. Statistical analysis

We used the Shapiro–Wilk test to analyze data depending on the normality of the distribution. Continuous variables in normal distributions are reported as mean [standard deviation (SD)], while skewed distributions are given as a median [interquartile range (IQR)]. Categorical variables are presented as number and percentage. Analysis of variance (ANOVA) tests or rank sum tests were used to compare continuous variables, while *χ*^2^ or Fisher’s exact tests were used to compare categorical variables across groups. Multivariate Cox regression analysis was utilized to assess the independent correlation between GNRI and 28 days mortality, with three models applied in the regression analysis. Model 1 was unadjusted, model 2 was only adjusted for age and sex, while model 3 included adjustments for model 2 as well as MI, CHF, COPD, AKI, diabetes, renal disease, malignant cancer, liver disease, RRT, MV, anion gap, BUN, RBC, WBC, platelets, hemoglobin, ALT, AST, INR, and SOFA. Restricted cubic spline models were used to investigate potential nonlinear correlations between GNRI levels and 28 days mortality risk with threshold analysis. Kaplan–Meier and log-rank analyses were employed to generate survival curves and present the cumulative risk of death within several groups of GNRI levels at admission. Subgroup analyses were carried out to confirm the reliability of the results, stratifying by age, comorbidities, and treatments.

Variables with missing values exceeding 20% were excluded from the analysis. For continuous variables with missing values less than 5%, the mean or median values were used to replace the missing values. The statistical software packages R (http://www.R-project.org, R Foundation) and Free Statistics software version 1.8 were adopted for all studies. The statistical analyses were performed using R software (http://www.R-project.org, R Foundation) and Free Statistics software (version 1.8). For all analyses, a two-tailed *p* < 0.05 was considered statistically significant.

## 3. Results

### 3.1. Baseline characteristics of patients

**Figure 1:**
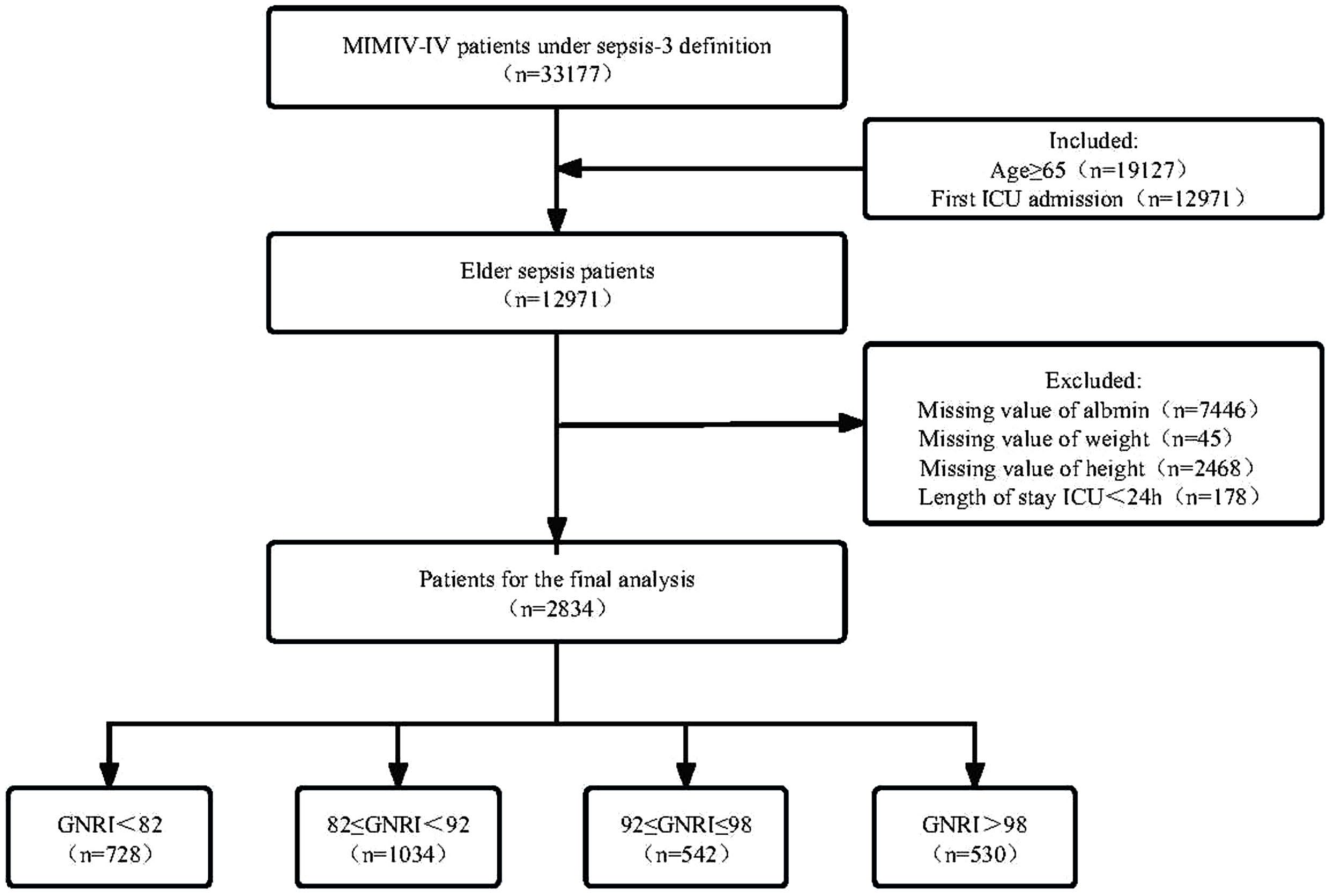
Flowchart of patient selection.

The MIMIC-IV database included 33,177 patients with sepsis-3 definition. After including only the first ICU admission of elderly patients and excluding key missing data, a total of 2,834 elderly patients with sepsis were included in this study based on the criteria established in Figure 1.

This population’s median age was 76.8 years (70.5–83.4). Among the patients, 55.9% were men (*n* = 1,585) and 44.1% were women (*n* = 1,249). Based on GNRI scores stratification, patients were divided into four groups: major risk group (GNRI<82; *n* = 728), moderate risk group (82 ≤ GNRI < 92, *n* = 1,034), low risk group (92 ≤ GNRI ≤ 98, *n* = 542), and no risk group (GNRI >98; *n* = 530). Table 1 listed the baseline characteristics of patients grouped by GNRI. Elderly septic patients exhibiting a combination of CHF, CPD, AKI, diabetes, and renal disease were found to have a higher incidence rate. Moreover, 94.7% had used mechanical ventilation (MV). Among laboratory indicators, compared to the no risk group, patients in the major risk group presented lower levels of aniongap, erythrocytes, platelets, and hemoglobin, and higher levels of BUN, WBC, ALT, AST, and INR. The severity of clinical conditions significantly increased as GNRI decreased. Patients in the major risk group had significantly higher 28 days mortality rates (42.3% vs. 23.2%, *p* < 0.001) and longer hospital stays [12.1 (6.3–20.6) vs. 9.2 (5.8–15.9), *p* < 0.001] compared to the no risk group. However, there was no statistically significant difference in the length of stay in the ICU (*p* = 0.614).

**Table 1:** Baseline characteristics of the study population according to GNRI.

Variables	Total (*n* = 2,834)	GNRI <82 (*n* = 728)	82 ≤ GNRI <92 (*n* = 1,034)	92 ≤ GNRI ≤ 98 (*n* = 542)	GNRI >98 (*n* = 530)	*p*
Age, years	76.8 (70.5, 83.4)	76.5 (70.3, 83.5)	77.0 (70.6, 83.8)	76.8 (70.2, 83.4)	76.4 (70.7, 82.5)	0.750
Gender, *n* (%)						0.051
Male	1,585 (55.9)	387 (53.2)	574 (55.5)	301 (55.5)	323 (60.9)	
Female	1,249 (44.1)	341 (46.8)	460 (44.5)	241 (44.5)	207 (39.1)	
*Comorbidities, n (%)*						
MI	737 (26.0)	146 (20.1)	290 (28)	164 (30.3)	137 (25.8)	<0.001
CHF	1,174 (41.4)	250 (34.3)	461 (44.6)	262 (48.3)	201 (37.9)	<0.001
COPD	860 (30.3)	203 (27.9)	347 (33.6)	175 (32.3)	135 (25.5)	0.003
AKI	1,412 (49.8)	384 (52.7)	524 (50.7)	249 (45.9)	255 (48.1)	0.083
Diabetes	963 (34.0)	212 (29.1)	378 (36.6)	187 (34.5)	186 (35.1)	0.011
Renal disease	865 (30.5)	216 (29.7)	343 (33.2)	166 (30.6)	140 (26.4)	0.048
Malignant cancer	468 (16.5)	173 (23.8)	171 (16.5)	71 (13.1)	53 (10)	<0.001
Liver disease	163 (5.8)	63 (8.7)	55 (5.3)	18 (3.3)	27 (5.1)	<0.001
*Treatment, n (%)*						
RRT	381 (13.4)	116 (15.9)	139 (13.4)	71 (13.1)	55 (10.4)	0.042
MV	2,685 (94.7)	690 (94.8)	983 (95.1)	517 (95.4)	495 (93.4)	0.457
*Laboratory parameters*						
Aniongap, mg/dL	16.0 (14.0, 19.0)	15.0 (13.0, 19.0)	16.0 (14.0, 19.0)	16.0 (14.0, 19.0)	17.0 (14.2, 20.0)	<0.001
BUN, mg/dL	28.0 (18.0, 45.0)	32.0 (19.0, 51.0)	30.0 (20.0, 48.0)	25.0 (17.0, 41.0)	24.0 (17.0, 36.0)	<0.001
RBC, 10^9^/L	3.6 (3.1, 4.2)	3.3 (2.9, 3.8)	3.6 (3.1, 4.1)	3.9 (3.4, 4.3)	4.2 (3.7, 4.6)	<0.001
WBC, 10^9^/L	12.0 (8.2, 16.8)	13.1 (8.5, 18.6)	12.6 (8.5, 17.7)	11.2 (8.1, 15.5)	10.5 (7.9, 14.5)	<0.001
Platelets, 10^9^/L	198.0 (140.0, 274.0)	195.0 (127.8, 294.0)	192.5 (129.0, 276.0)	202.0 (150.0, 266.0)	201.5 (157.2, 257.8)	0.246
Hemoglobin, g/dL	11.0 (9.4, 12.6)	9.9 (8.5, 11.3)	10.7 (9.2, 12.2)	11.5 (10.1, 12.9)	12.6 (11.1, 13.9)	<0.001
ALT, U/L	27.0 (16.0, 59.5)	30.0 (15.0, 71.0)	29.0 (16.0, 65.0)	24.0 (16.0, 59.0)	24.0 (16.0, 39.2)	0.007
AST, U/L	41.0 (25.0, 95.0)	46.0 (25.0, 117.0)	44.0 (26.0, 106.0)	39.0 (24.0, 92.0)	33.0 (23.0, 65.0)	<0.001
INR	1.3 (1.1, 1.6)	1.4 (1.2, 1.8)	1.3 (1.2, 1.6)	1.2 (1.1, 1.5)	1.2 (1.1, 1.4)	<0.001
*Scoring*						
SOFA	3.0 (2.0, 5.0)	4.0 (3.0, 5.0)	3.0 (2.0, 5.0)	3.0 (2.0, 4.0)	3.0 (2.0, 4.0)	<0.001
SAPS II	46.0 (37.0, 56.0)	51.0 (42.0, 60.0)	46.0 (38.0, 55.0)	43.0 (36.0, 52.0)	41.0 (33.2, 51.0)	<0.001
*Outcomes*						
LOS ICU, day	4.9 (2.6, 9.0)	4.9 (2.6, 9.7)	4.8 (2.6, 9.1)	4.9 (2.4, 8.3)	4.8 (2.7, 8.8)	0.614
LOS hospital, day	10.5 (6.1, 17.5)	12.1 (6.3, 20.6)	10.7 (6.4, 16.9)	9.5 (6.1, 15.4)	9.2 (5.8, 15.9)	<0.001
28 days mortality, *n* (%)	881 (31.1)	308 (42.3)	321 (31)	129 (23.8)	123 (23.2)	<0.001

GNRI, geriatric nutritional risk index; MI, myocardial infarction; CHF, congestive heart failure; COPD, chronic obstructive pulmonary disease; AKI, acute kidney injury; RRT, renal replacement therapy; MV, mechanical ventilation; BUN, blood urea nitrogen; RBC, red blood cell; WBC, white blood cell; ALT, alanine transaminase; AST, aspartate aminotransferase; INR, international normalized ratio; SOFA, sequential organ failure assessment; SAPS II, simplified acute physiology score II; LOS, length of stay.

### 3.2. Multivariable Cox regression analysis

In this study, we designed three models to examine the effect of GNRI on 28 days mortality in elderly septic patients in the ICU using a multivariate Cox regression model (Table 2). In the unadjusted model (model 1) where GNRI was treated as a continuous variable, we found a negative correlation between GNRI and 28 days mortality. Specifically, each one-unit increase in GNRI corresponded to a 3% reduction in the risk of 28 days mortality (HR: 0.97, 95% CI: 0.97–0.98, *p* < 0.001). Both the fully adjusted model (model 3) and the slightly adjusted model (model 2) maintained this connection.

**Table 2:** Multivariable Cox regression to assess the association of GNRI with 28 days mortality.

Variable	Model 1		Model 2		Model 3	
	HR (95% CI)	*p*-value	HR (95% CI)	*p*-value	HR (95% CI)	*p*-value
GNRI	0.97 (0.97–0.98)	<0.001	0.97 (0.97–0.98)	<0.001	0.97 (0.97–0.98)	<0.001
GNRI <82	1 (Ref)		1 (Ref)		1 (Ref)	
82 ≤ GNRI < 92	0.67 (0.57–0.78)	<0.001	0.68 (0.58–0.8)	<0.001	0.66 (0.56–0.78)	<0.001
92 ≤ GNRI ≤ 98	0.5 (0.4–0.61)	<0.001	0.53 (0.43–0.65)	<0.001	0.52 (0.42–0.65)	<0.001
GNRI >98	0.48 (0.39–0.6)	<0.001	0.52 (0.42–0.65)	<0.001	0.5 (0.4–0.63)	<0.001
*p* for trend	0.77 (0.72–0.82)	<0.001	0.79 (0.74–0.84)	<0.001	0.78 (0.72–0.84)	<0.001

Model 1: Non-adjusted model. Model 2: Adjust for age and gender. Model 3: Model 2 + MI, CHF, COPD, AKI, diabetes, renal disease, malignant cancer, liver disease, RRT, MV, aniongap, BUN, RBC, WBC, platelets, hemoglobin, ALT, AST, INR, SOFA.

When GNRI was considered as a categorical variable, the unadjusted model (model 1) revealed a significant negative correlation between the GNRI category and 28 days mortality. The death rates for the major risk groups were greater than those for the low or no risk groups (major vs. moderate: HR = 0.67, 95% CI: 0.57–0.78; major vs. low: HR = 0.5, 95% CI: 0.4–0.61; major vs. no risk: HR = 0.48, 95% CI: 0.39–0.6; *p* for trend <0.001). In the fully adjusted model (model 3), the multivariate hazard ratios (HRs) for 28 days mortality were 0.66 (95% CI, 0.56–0.78), 0.52 (95% CI, 0.42–0.65), and 0.5 (95% CI, 0.4–0.63) for the moderate, low, and no risk groups, respectively (*p* < 0.001). Consistent results were obtained across all models, demonstrating the robustness of the statistical outcomes.

### 3.3. Restricted cubic splines analysis

**Figure 2:**
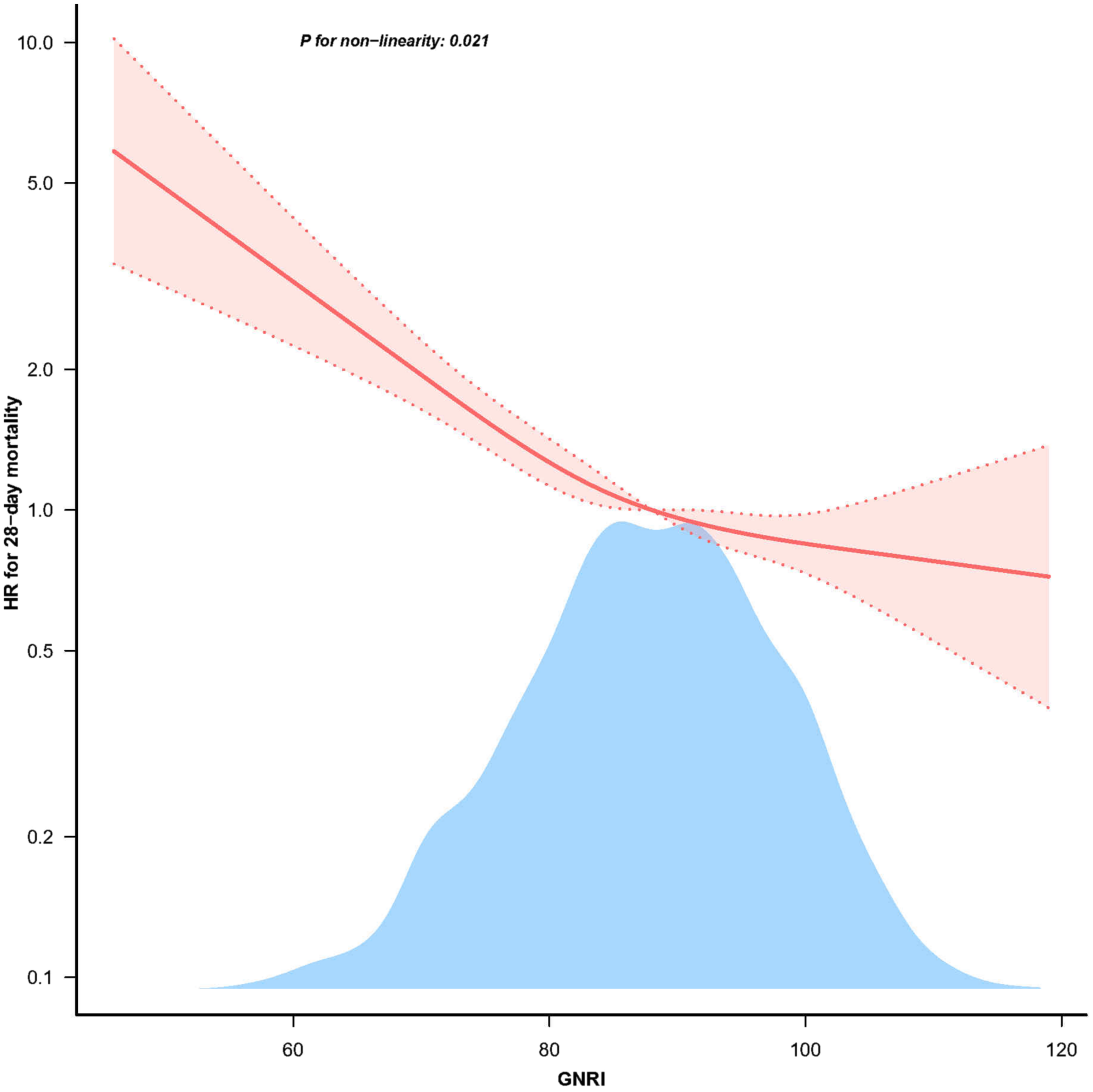
The non-linear relationship between GNRI and 28 days mortality in patients with elder sepsis.

The restricted cubic spline analysis demonstrated a non-linear relationship between GNRI and 28 days mortality after adjusting for related confounding factors (Figure 2, *p* = 0.021). According to the two-piecewise linear regression models, the GNRI for hospital and ICU mortality peaked at 98.1. A significant negative correlation between GNRI levels and 28 days mortality was seen on the left side of the inflection point (HR = 0.967, 95% CI: 0.959–0.974; *p* < 0.001), with a discernible decline in 28 days mortality as GNRI levels increased. On the right side of the inflection point, however, there was no correlation between GNRI levels and 28 days mortality (Table 3).

**Table 3:** Threshold effect analysis of GNRI on 28 days mortality.

Item	HR (95% CI)	*p*
Inflection point	98.1 (93.8, 102.4)	
Slope 1	0.967 (0.959, 0.974)	<0.001
Slope 2	1.043 (0.984, 1.106)	0.1549
Likelihood ratio test		0.023

### 3.4. Kaplan–Meier survival curve analysis

**Figure 3:**
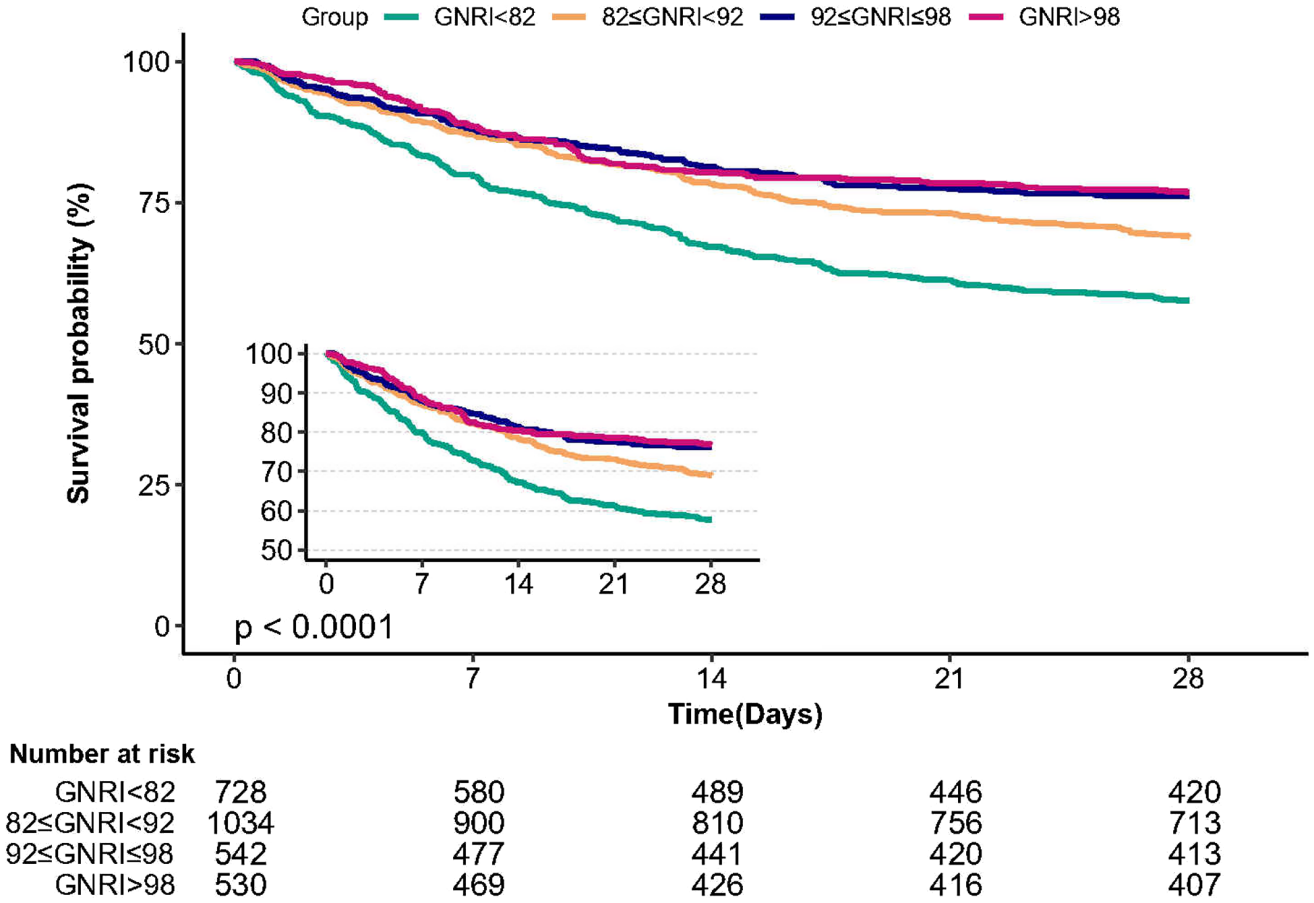
Kaplan–Meier survival curve for the cumulative hazard of 28 day mortality.

The Kaplan–Meier survival curve analysis demonstrated that patients in the major risk group (GNRI <82) had the lowest 28 days survival rate compared to the other three groups, with a significant decrease observed with decreasing GNRI (Figure 3, *p* < 0.0001).

### 3.5. Subgroup analyses

**Figure 4:**
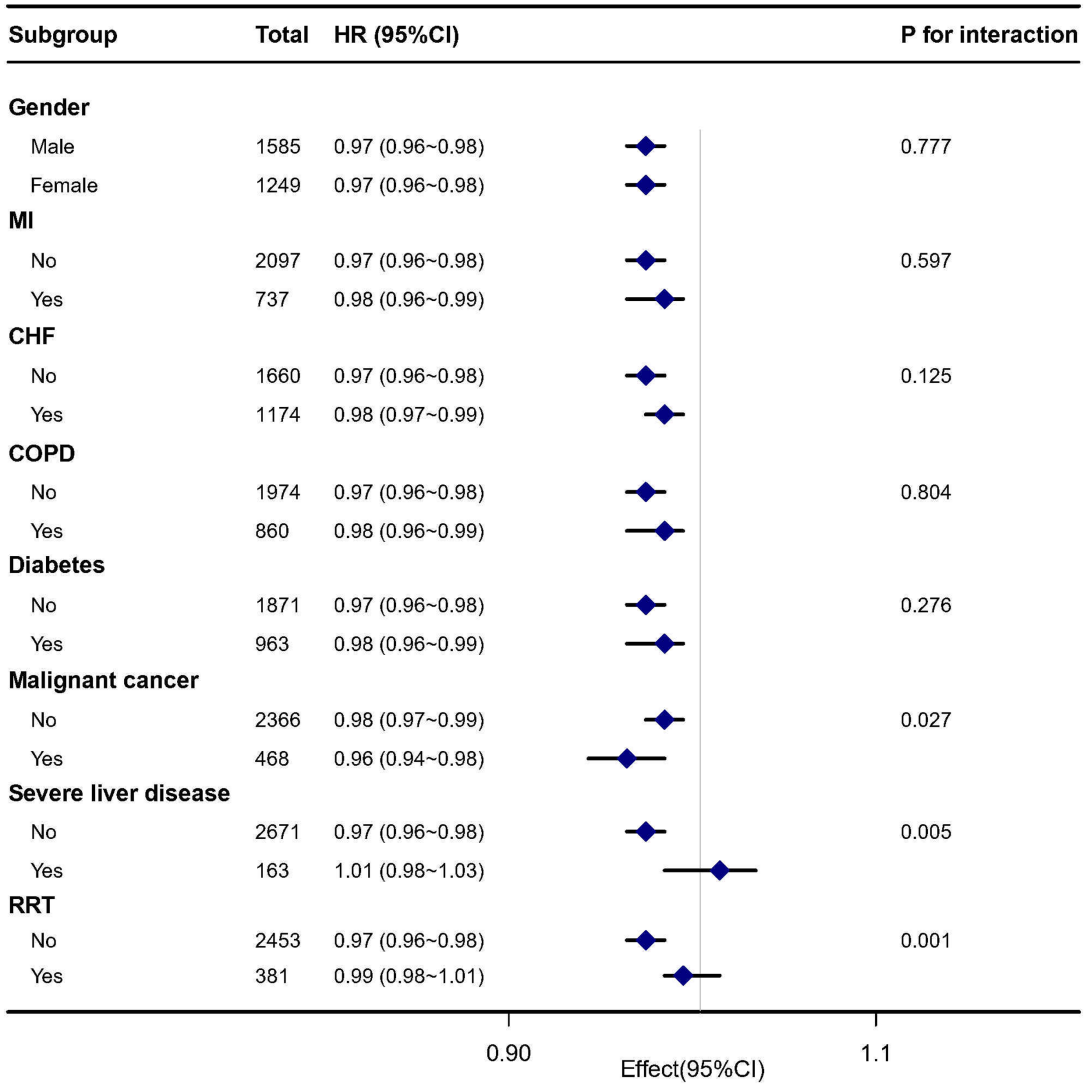
Subgroup analysis of the association between GNRI and 28 days mortality in elderly septic patients.

Figure 4 indicates whether the correlation between GNRI and 28 days mortality in patients with sepsis was stable across subgroups. Several subgroup analyses were conducted according to gender, MI, CHF, COPD, diabetes, malignant cancer, severe liver disease, RRT. After adjusting for multivariates, we found significant interactions detected in the subgroups with malignant cancer (*p* for interaction = 0.027), severe liver disease (*p* for interaction = 0.005), and RRT (*p* for interaction = 0.001). Specifically, a stronger association was observed between GNRI levels and 28 days mortality in elderly septic patients with malignant cancer (HR = 0.96, 95% CI: 0.94–0.98), severe liver disease (HR = 1.01, 95% CI: 0.98–1.03), and RRT (HR = 0.99, 95% CI: 0.98–1.01).

## 4. Discussion

Concerning elderly sepsis patients, the objective of this retrospective cohort study was to look into the relationship between GNRI levels at admission and 28 days mortality. We observed a strong correlation between the GNRI and 28 days mortality in this population. Particularly, compared to the low-risk group, participants in the high-risk group had considerably greater fatality rates and longer hospital stays. Multivariate Cox regression analyses consistently yielded similar results, reinforcing the robustness of our findings. Additionally, the restricted cubic spline analysis indicated a nonlinear relationship between GNRI and 28 days mortality. Subgroup analyses demonstrated significant interactions with malignancy, severe liver disease, and receipt of RRT. These findings imply that the influence of GNRI on mortality might be particularly significant in elderly septic patients with these underlying conditions.

Many countries are currently experiencing the phenomenon of aging societies, characterized by a significant increase in the elderly population (≥80 years), resulting in a consequent rise in ICU admissions of elderly patients (20). While previous studies have explored the association between GNRI and elderly patients with acute kidney injury (21), multiple organ dysfunction syndromes (22), acute respiratory failure (23), and trauma (24), our study is the first to examine the relationship between GNRI and the prognosis of elderly septic patients in ICU. Elderly patients suffering from sepsis face elevated mortality rates, often exacerbated by diagnostic delays attributed to the complexities of their conditions (25). Research indicates that mortality rates increase in septic patients with advancing age, with individuals aged ≥80 years exhibiting a higher mortality rate compared to those aged 65–79 years (25). Our study revealed that lower GNRI levels in older septic patients were linked to the extended in-hospital length of stay and increased 28 days mortality, emphasizing the significance of nutritional screening. However, there was no observed association between GNRI and duration of ICU stay, which aligns with the findings of a previous study by Kaddoura et al. (26). It is noteworthy that our study employed a retrospective design, limiting our ability to determine the underlying mechanism of this effect. However, the lack of a significant relationship between GNRI and ICU LOS could potentially be attributed to the relatively short average duration of ICU stay, which was 4.9 days. Prior studies (21–24) have explored the prognostic significance of GNRI in specific ICU populations. However, the optimal GNRI cutoff values for elderly septic patients as a whole remain unclear. We investigated the association between GNRI levels and 28 days mortality in elderly patients with sepsis, utilizing a restricted three-sample bar and smoothing curve analysis. According to our research, there is an L-shaped link between continuous GNRI levels and the probability of 28 days death, which is in line with the findings of Peng et al. (27). Furthermore, our study revealed the predictive power of GNRI in forecasting in-hospital mortality among elderly septic patients. Elderly septic patients, underscoring the importance of early detection and intervention for malnutrition. This aligns with the conclusions drawn from a previous study conducted by Peng et al. (27). Consequently, elderly septic patients who present malnutrition risk upon ICU admission tend to experience prolonged hospital stays. Our subgroup analysis revealed a more pronounced association between GNRI and short-term mortality in patients with malignancy or severe liver disease. Existing evidence from two meta-analyses indicates that low GNRI levels are significantly linked to increased mortality risk in patients with gastrointestinal malignancies (28) and lung cancer (29). Furthermore, several research has shown the predictive usefulness of GNRI in hepatocellular carcinoma (30), renal cancer (31), and bladder cancer (32). In summary, these findings underscore the clinical utility of GNRI as a nutritional assessment tool for elderly individuals diagnosed with cancer.

In addition, our findings provide a basis for further exploration of nutrition-based interventions to improve the prognosis and quality of life of elderly sepsis patients. We can think about applying nutritional therapies for elderly sepsis patients based on the correlation between the GNRI and 28 days mortality. To help in patient recovery and enhance long-term results, this may entail offering foods with a high nutritional value, supplementing nutrients, and maintaining good nutritional status. In the treatment of sepsis, timely identification and control of infection sources are crucial. It is important to administer appropriate antibiotic therapy and surgical interventions for different infection sources to reduce the severity of infection and enhance treatment efficacy. Therefore, physicians should develop individualized infection source control strategies based on specific patient conditions. To validate the benefits of these interventions, future researchers may consider conducting prospective intervention trials.

Our research has some limitations. Firstly, because it is an observational study, we are inevitably confronted with potential confounding factors that cannot be avoided. To test the validity of our findings, we systematically corrected for confounding variables and used subgroup analysis. Secondly, we can only observe associations and cannot assess causal correlations due to the inherent limits of observational studies. In order to confirm the association between GNRE and elderly sepsis prognosis, additional high-quality prospective studies are required. We were able to account for quantifiable confounders but not for unmeasurable ones. Lastly, it might be challenging to extrapolate our findings to other groups because our study was constrained to the MIMIC database and a single nation. Further medical research with larger sample sizes and greater levels it might be challenging to extrapolate our findings.

## 5. Conclusion

In conclusion, our research showed that GNRI can accurately predict 28 days death in elderly sepsis patients who are diagnosed in the ICU. Further investigation is required to gain a better understanding of the underlying mechanisms and potential strategies to enhance outcomes in this study.

## Data availability statement

The raw data supporting the conclusions of this article will be made available by the authors, without undue reservation.

## Ethics statement

Ethical review and approval was not required for the study on human participants in accordance with the local legislation and institutional requirements. Written informed consent for participation was not required for this study in accordance with the national legislation and the institutional requirements.

## Author contributions

LL: Writing – original draft, Writing – review & editing. XL: Writing – review & editing. SQ: Writing – review & editing. DH: Writing – review & editing.

## Funding

The author(s) declare financial support was received for the research, authorship, and/or publication of this article. This study was financed by the Project on Enhancement of Basic Research Ability of Young and Middle-aged Teachers in Guangxi Universities and Colleges, No. 2023KY0131. The corresponding author Debin Huang, received the funding in 2023.

## Conflict of interest

The authors declare that the research was conducted in the absence of any commercial or financial relationships that could be construed as a potential conflict of interest.

## Publisher’s note

All claims expressed in this article are solely those of the authors and do not necessarily represent those of their affiliated organizations, or those of the publisher, the editors and the reviewers. Any product that may be evaluated in this article, or claim that may be made by its manufacturer, is not guaranteed or endorsed by the publisher.
